# NR4A1 (Nur77) Mediates Thyrotropin-Releasing Hormone-Induced Stimulation of Transcription of the Thyrotropin β Gene: Analysis of TRH Knockout Mice

**DOI:** 10.1371/journal.pone.0040437

**Published:** 2012-07-09

**Authors:** Yasuyo Nakajima, Masanobu Yamada, Ryo Taguchi, Nobuyuki Shibusawa, Atsushi Ozawa, Takuya Tomaru, Koshi Hashimoto, Tsugumichi Saito, Takafumi Tsuchiya, Shuichi Okada, Tetsurou Satoh, Masatomo Mori

**Affiliations:** Department of Medicine and Molecular Science, Gunma University Graduate School of Medicine, Maebashi, Japan; Cardiff University, United Kingdom

## Abstract

Thyrotropin-releasing hormone (TRH) is a major stimulator of thyrotropin-stimulating hormone (TSH) synthesis in the anterior pituitary, though precisely how TRH stimulates the TSHβ gene remains unclear. Analysis of TRH-deficient mice differing in thyroid hormone status demonstrated that TRH was critical for the basal activity and responsiveness to thyroid hormone of the TSHβ gene. cDNA microarray and K-means cluster analyses with pituitaries from wild-type mice, TRH-deficient mice and TRH-deficient mice with thyroid hormone replacement revealed that the largest and most consistent decrease in expression in the absence of TRH and on supplementation with thyroid hormone was shown by the TSHβ gene, and the NR4A1 gene belonged to the same cluster as and showed a similar expression profile to the TSHβ gene. Immunohistochemical analysis demonstrated that NR4A1 was expressed not only in ACTH- and FSH- producing cells but also in thyrotrophs and the expression was remarkably reduced in TRH-deficient pituitary. Furthermore, experiments *in vitro* demonstrated that incubation with TRH in GH4C1 cells increased the endogenous NR4A1 mRNA level by approximately 50-fold within one hour, and this stimulation was inhibited by inhibitors for PKC and ERK1/2. Western blot analysis confirmed that TRH increased NR4A1 expression within 2 h. A series of deletions of the promoter demonstrated that the region between bp -138 and +37 of the TSHβ gene was responsible for the TRH-induced stimulation, and Chip analysis revealed that NR4A1 was recruited to this region. Conversely, knockdown of NR4A1 by siRNA led to a significant reduction in TRH-induced TSHβ promoter activity. Furthermore, TRH stimulated NR4A1 promoter activity through the TRH receptor. These findings demonstrated that 1) TRH is a highly specific regulator of the TSHβ gene, and 2) TRH mediated induction of the TSHβ gene, at least in part by sequential stimulation of the NR4A1-TSHβ genes through a PKC and ERK1/2 pathway.

## Introduction

Thyrotropin-releasing hormone (TRH) was originally isolated as the first hypothalamic hormone [1], [2] and a major stimulator of the secretion of thyrotropin (TSH) from the anterior pituitary gland [3]. Subsequently, TRH was also found to promote production of TSH in part by stimulating transcription of the TSHβ and α genes. TRH binds to its receptor in the anterior pituitary and activates phospholipase C, leading to calcium mobilization and protein kinase C activation [4]–[8] and also stimulation of the MAPK pathway [9], [10]. The actions of these intracellular signaling pathways ultimately lead to an increase in transcription of the TSHβ and α genes [11], [12]. However, precisely how TRH mediates transcription of the TSHβ gene *in vivo* still remains unclear.

A pituitary-specific transcription factor, Pit1, was first postulated as a candidate protein that influences TRH-induced stimulation of the TSHβ gene. Pit1 which contains two transactivation domains termed the POU-specific domain and POU homeo domain is expressed in somatotrophs, lactotrophs and thyrotrophs, and is critical for the development of pituitary thyrotrophs [13]. In fact, a patient with a mutation of the Pit1 gene exhibited TSH, PRL- and GH- deficiency [14], [15]. Pit1 has also been reported to be important for regulation of the TSHβ gene by TRH [13]. TRH-dependent phosphorylation of Pit1 has been suggested to increase Pit1-binding to low-affinity TSHβ promoter-binding sites, and overexpression of a mutant Pit1 containing the DNA-binding domain but lacking the major transactivation domain substantially blocked the TRH-induction of the TSHβ promoter activity in GH3 cells [16]. Therefore, TRH may exert its function by changing the state of the Pit1 protein.

The second candidate for a protein involved with TRH-induced stimulation of the TSHβ gene is GATA2 [17]. GATA2 belongs to a subtype of transcription factors, the GATA family, that binds through its Zn finger domain with the GATA-responsive element (GATA-RE), which has high homology among all GATA family members [18]. GATA2 is expressed in thyrotrophs and gonadotrophs in the pituitary [19]. It has been reported that TRH enhanced GATA2- dependent activation of the TSHβ promoter and that this stimulation was abolished by an amino-acid substitution of the GATA2-Zn finger domain or a mutation of the GATA-responsive element of the TSHβ gene. In addition, an recent EMSA study by Oba et al revealed that TRH increased the DNA-binding capacity of GATA2 on the gene [20].

We generated TRH-deficient mice using homologous recombination in embryonic stem cells [21]. These mice show characteristic phenotypes, including tertiary hypothyroidism and mild hyperglycemia. The basal serum TSH level was unexpectedly elevated, and the result of the TRH test suggested that the secreted TSH had reduced biological activity. An ontogeny based analysis of these mice demonstrated that there was no requirement for TRH in the development of embryonic thyrotrophs in the pituitary, but TRH was required for the maintenance of the normal function of pituitary thyrotrophs [22].

NR4A1 (also known as Nur77, NGFI-B or TR3) belongs to a superfamily of orphan nuclear receptors and was originally isolated as an immediate-early response gene induced by a nerve growth factor in a pheochromocytoma cell line, PC12 [23]. NR4A1 is also regulated by many physiological stimuli including growth factors, inflammatory signals and hormones, and implicated in a wide range of important biological processes including apoptosis, brain development, glucose metabolism, and vascular remodeling [24]–[27]. Expression of NR4A1 has also been identified in several endocrine organs including the anterior pituitary, ovary, adrenal gland, and testis [28]–[35]. Several investigators have demonstrated that NR4A1 plays an important role in the function of the hypothalamo-pituitary-adrenal (HPA) axis and hypothalamo-pituitary-gonadal (HPG) axis [32], [33], [36], [37]. However, there is no report regarding the involvement of NR4A1 in the hypothalamo-pituitary-thyroid axis, since there has been no appropriate animal model to use.

Pit1 is necessary but not sufficient for basal transcription of the TSHβ gene, in fact, the basal activity of the TSHβ promoter was minimally stimulated when co-transfected with a mouse Pit1 in the thyrotroph cell lines αTSH and TtT-97 [38]. In addition, several studies indicated that TRH-induced activation of the TSHß gene requires both PKC and MAPK pathways [4]–[10], [12]. TRH-induced stimulation of GATA2-activation of the TSHβ promoter depends on PKC but not MAPK in GH3 cells [20]. Therefore, there may be additional factors involved with TRH-induced stimulation of the TSHβ gene *in vivo.*

In the present study, to elucidate the mechanisms underlying TRH-induced stimulation of the TSHβ gene *in vivo*, we first examined the role of TRH in the secretion and synthesis of TSH by measuring serum TSH and pituitary TSH mRNA levels in different thyroid status in TRH knockout mice. We then attempted to determine factors related to action of TRH on the TSHβ gene through expression profiling of pituitary mRNAs using wild-type and TRH-deficient pituitary supplemented with thyroid hormone. We discovered that an orphan nuclear receptor, NR4A1, played a critical role in TRH-mediated stimulation of the pituitary TSHβ gene *in vivo*.

## Results

### TRH Regulates the Basal Activity and Responsiveness to Thyroid Hormone of the TSHβ Gene In vivo

**Figure 1:**
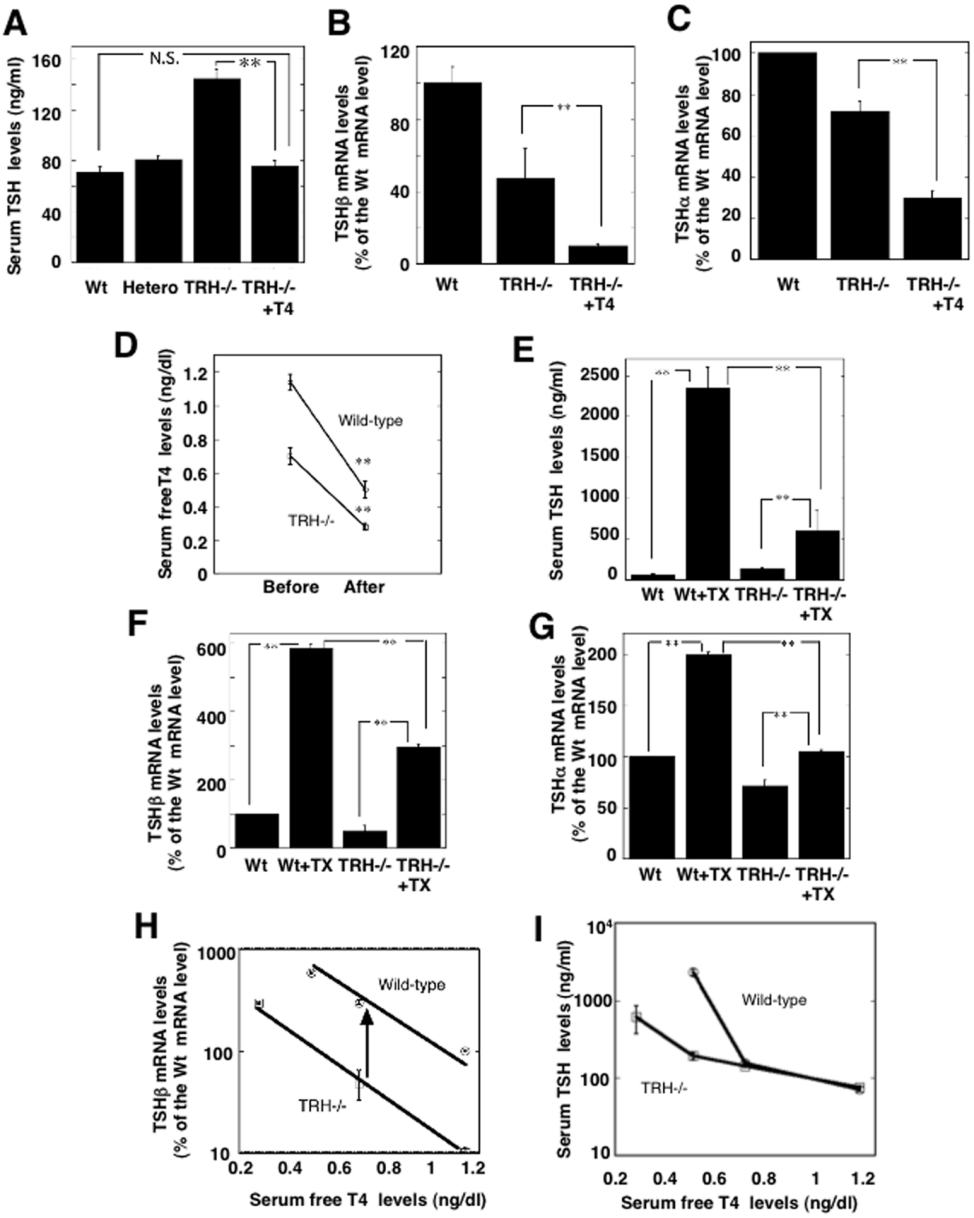
The hypothalamic-pituitary-thyroid axis in TRH deficient mice. A) Serum TSH levels were measured in wild-type mice (Wt), TRH knockout mice (TRHKO) heterozygotes (Hetero) and homozygotes (TRH−/−) and TRHKO supplemented with thyroid hormone (TRH−/− + T4). TRHKO (n = 7) were supplemented with thyroid hormone by the daily injection of T4 (1.5 μg/100 g body weight) for 14 days to be euthyroid (n = 6). The serum TSH level in TRH−/−+T4 mice was similar to that in the wild-type (n = 7) and heterozygous mice (n = 6). Values are represented as the mean ± SEM. **, *p*<0.01; *, N.S., not significant. B) TSHβ mRNA levels were measured in pituitaries of the wild-type mice, TRHKO, and TRHKO supplemented with thyroid hormone (TRH−/−+T4). The TSHβ mRNA level in TRH−/− was decreased to about 50% of that in the wild-type, and that of TRH−/−+T4 mice was further decreased to about 10% (n = 3, *p<*0.01). **, *p*<0.01. C) TSHα mRNA levels showed a similar profile to the TSHβ mRNA level shown in Fig 1B. However, the change in TSHβ mRNA was more profound than that in TSHα mRNA. **, *p*<0.01. D) Two weeks after thyroidectomy, serum free T4 levels were significantly reduced in both the wild-type mice (n = 6) and TRHKO mice (TRH−/−) (n = 5). The serum T4 levels were significantly lower in TRH−/− mice than wild-type mice. **, *p*<0.01. E) Reflecting hypothyroidism induced by thyroidectomy (TX), the serum TSH level in the wild-type mice showed an approximately 25-fold increase (Wt+TX, n = 6), but TRHKO mice (TRH−/−) showed only about a 6-fold increase (TRH−/−+TX, n = 6). **, *p*<0.01. F) The TSHβ mRNA levels in thyroidectomized wild-type mice (Wt+TX) were significantly increased after two weeks to approximately 6-fold the control value (Wt) (n = 3). However, the TRHKO mice (TRH−/−) showed a lesser increase (TRH−/−+TX)(n = 3). **, *p*<0.01; G) A similar profile was observed in TSHα mRNA levels in the pituitary. But a milder effect was observed compared to those of TSHβ mRNA levels. **, *p*<0.01; n = 3. H) Correlation between serum thyroid hormone levels and the corresponding pituitary TSHβ mRNA levels. Circles represent values for the wild-types and the squares, those for TRH knockout mice (TRH−/−). When comparing the slopes of the curve, it was demonstrated that TRH altered the basal activity and responsiveness of the TSHβ gene to thyroid hormone. The arrow indicates the effect of TRH, taking approximately 80% responsibility for the value of the TSHβ mRNA level in the wild-type pituitary. I) Correlation between serum thyroid hormone levels and the corresponding serum TSH levels. In mild hypothyroidism, the serum TSH levels in TRHKO mice (TRH−/−) were similar to those in the wild-type mice. However a lack of TRH induced a marked impairment of the serum TSH level in severe hypothyroid status.

As previously reported, the serum TSH levels in homozygotes of TRH knockout mice (TRHKO) (143.9±7.9 ng/ml, n = 7) were slightly but significantly higher that those in the wild-type (71.3±4.1 ng/ml, n = 7, *p<*0.01) and heterozygous mice (81.2±2.5, n = 6, *p<*0.01). Previous studies suggested that the elevated serum TSH levels in TRHKO reduced the biological activity [22], [39]. In order to examine the feedback system of the hypothalamo-pituitary-thyroid axis in TRHKO, we first measured serum TSH levels in euthyroid TRHKO given daily injections of T4. The decreased serum thyroid hormone level normalized (1.92±0.05 ng/ml, n = 6) following the daily subcutaneous injection of thyroid hormone (T4, 1.5 μg/100 g body weight) for 14 days. In these euthyroid TRHKO, the elevated serum TSH levels reverted to normal (75.8±3.7 ng/ml, n = 6). These results suggested the serum TSH level to be normally regulated by thyroid hormone in the absence of TRH (Fig. 1A). To determine the mechanism of the normalization of the serum TSH level in euthyroid TRHKO, we next measured TSHβ and α mRNA levels in the pituitary by real-time PCR with TaqMan probes. Although serum TSH levels in the intact TRHKO were significantly elevated, pituitary TSHβ and α mRNA levels were paradoxically decreased to 47.4±16.2% and 71.8±5.0% of those in the control wild-type mice, respectively. In contrast to the change of serum TSH level in the euthyroid TRHKO, both TSHβ and α mRNA levels were further reduced to 10.1±1.1% and 30.1±3.0% of the control values (Fig.1B and C). Furthermore, a predominant reduction was observed in the TSHβ mRNA level as compared to the TSHα mRNA level. These results suggested that the normalization of the serum TSH level in TRHKO by thyroid hormone may be, at least in part, due to a decrease in the synthesis of TSH in the pituitary. Since there were no reports of thyroidectomy in mice, we first established a technique using dissection microscopy [40]. The thyroidectomy for mice was basically the same as that for rats, except for the use of microscopy. The success rate of this procedure was approximately 70%, and the rest of the mice died due to respiratory failure with damage to the phrenic nerve. In the wild-type mice, thyroidectomy caused a significant decrease in the serum thyroid hormone levels from 1.14±0.55 ng/ml (n = 6) to 0.50±0.11 ng/ml (n = 6) within two weeks. Although the serum T4 level in intact TRHKO was approximately 60% of that in the wild-type mice (0.7±0.05 ng/ml, n = 7), thyroidectomy further decreased these levels to 0.28±0.02 ng/ml (n = 5)(Fig. 1D). Reflecting the hypothyroid status, the serum TSH level of the wild-type mice (64.2±4.4 ng/ml) was significantly increased approximately 25-fold of the control (2349.5±255.5 ng/ml) after two weeks. In contrast, although the serum TSH level was higher in the intact TRHKO than wild-type mice, the increase was insufficient (611±240 ng/ml, n = 6) compared to that in the wild-type control (Fig 1E).

To evaluate the mechanism of the insufficient increase in the serum TSH levels in TRHKO in response to hypothyroidism induced by thyroidectomy, we measured TSHβ and α mRNA levels in the TRHKO pituitary and compared them to those in the wild-type. As expected thyroidectomy caused a significant increase in the TSHβ mRNA level, to approximately 6-fold (584.9±13.4%) that in the wild-type pituitary. Similarly, the TSHα mRNA level was increased to 199.0±2.4%. However, the increase of the TSHβ mRNA level in TRHKO was only approximately 3 fold (296±7.8%), and similarly, the TSHα mRNA level was increased to 105±1.3% of the control (Fig 1F, and G).

The experimental hypothyroidism induced by MMI treatment with regular chow that contained iodine in a previous study induced a milder hypothyroidism than that due to thyroidectomy [22]. The serum thyroid hormone level in the MMI-induced hypothyroid wild-type mice was similar to that in the simple TRHKO. The treatment with MMI increased the TSHβ mRNA level to 300.0±15.5%. To analyze the correlation between the serum thyroid hormone levels and the corresponding TSHβ mRNA levels and serum TSH levels, all the data was plotted in. As shown in Fig 1H, it is clear that each pituitary TSHβ mRNA level corresponding to the serum thyroid hormone level in the TRHKO pituitary was significantly lower than those of the wild-type controls. Furthermore, when compared to the slope of the correlation curve, the slope for the TRHKO was almost parallel to that in the wild-type mice, suggesting that TRH affected the basal activity and the responsiveness of the TSH gene to thyroid hormone. When the TSHβ mRNA level at the middle of this curve was calculated, approximately 80% (83,7% at serum T4, 0.7 ng/ml) depended on the presence of TRH.

It is important to note that although the serum TSH levels in TRHKO with mild hypothyroidism were similar to those in the wild-type mice, the lack of TRH impaired the raising of serum TSH levels, particularly in mice with severe hypothyroidism, as shown in Fig 1I.

### cDNA Microarray Analysis of the TRH-deficient Pituitary with and without Thyroid Hormone Replacement

**Figure 2:**
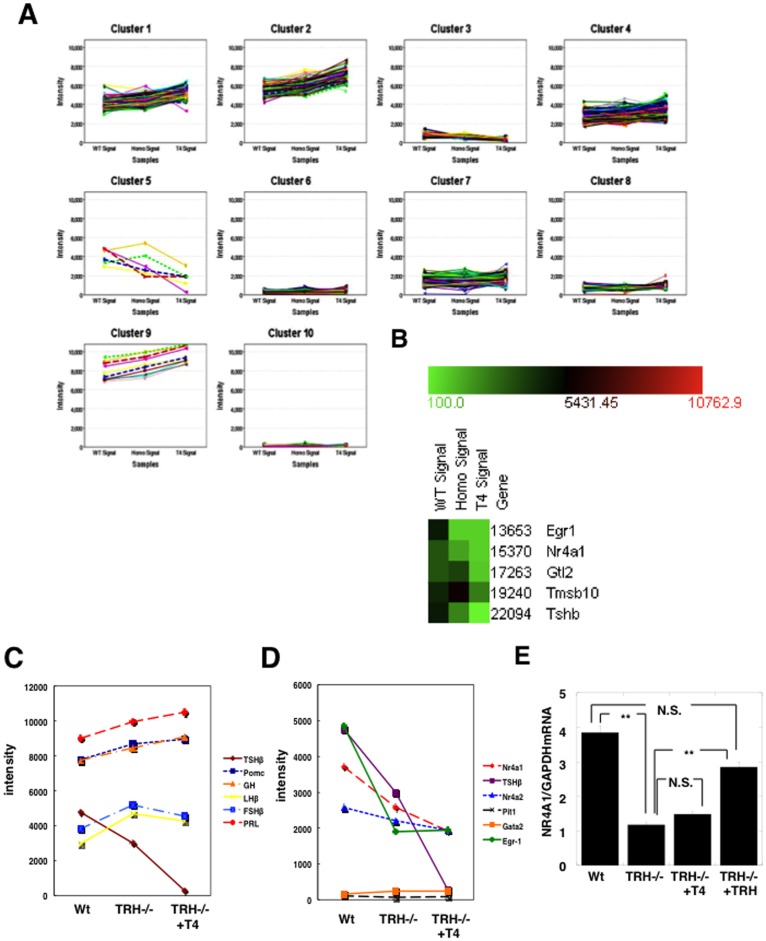
Microarray and K-means cluster analyses of genes regulated by TRH and by thyroid hormone in TRH-deficient pituitary. RNA samples from pituitaries of wild-type, TRHKO (TRH−/−), and euthyroid TRHKO mice with thryroid hormone replacement (TRH−/−+T4) were subjected to a whole-genome microarray using Affymetrix Mouse Gene chip 320 2.0 Array. After exclusion of absent signals (less than 100 signals), 10445 out of 45102 genes expressed in the TRHKO pituitary were subjected to the cluster analysis using a K-means algorithm. Hierarchical cluster analysis was carried out using a computer program, Genowiz™ 3.2. A) Genes were divided into ten hierarchical clusters according to changes and profiles among three groups, wild-type (WT signal), TRH−/− (Homo signal), and TRH−/− + T4 (T4 signal). B) In addition to the TSHβ genes, cluster 5 contained the Egr-1, NR4A1, Glt2, and Tmsb10 genes. C) mRNA levels of other anterior pituitary hormones including POMC, GH, LHβ, FSHβ, and prolactin (PRL) were not altered among the three groups, suggesting the expression to be TRH-independent. D) Profiles of the NR4A1, NR4A2, Egr-1, Pit1 and GATA2 genes are depicted. Expression of NR4A1, NR4A2 and Egr-1 might be TRH-dependent. E) Confirmation of results of the microarray analysis of NR4A1 mRNA levels in the TRH-deficient pituitary by real-time PCR. TRHKO mice (TRH−/−, n = 3) were supplemented with thyroid hormone by the daily injection of T4 (TRH−/−+T4, n = 3) and with TRH by 1 mg/kg body weight/day with an Alzet pomp for 7 days (TRH−/−+TRH n = 3). Total RNA obtained from these pituitaries was subjected to real-time PCR. The NR4A1 mRNA level in the TRHKO pituitary was decreased to about 25% of that of the wild-type. The decreased NR4A1mRNA level was not altered by thyroid hormone replacement, but completely reversed to the normal level by TRH administration, suggesting expression of NR4A1 mRNA in the pituitary to be TRH-, but not thyroid hormone-dependent.

In order to identify the factors involved with regulation of the pituitary TSH gene by TRH, we examined the gene expression in TRH-deficient pituitary with or without thyroid hormone replacement. Total RNAs were extracted from the pituitary of wild-type (WT signal), TRHKO (Homo Signal), and euthyroid TRHKO with thyroid hormone replacement (T4 signal) (TRHKO +T4∶1.5 μg T4/100 g body weight, subcutaneously for 14 days) and subjected to a cDNA microarray analysis using the Affymetrix Mouse Gene Chip 320 2.0 Array. After exclusion of absent signals (less than 100 signals), 10445 probes were expressed out of 45102 probes and subjected to a cluster analysis using a K-means algorithm. As shown in Fig 2A, it was classified into ten clusters according to profiles of changes among three groups and the expression level of the gene. Most genes belonging to clusters other than cluster 5 showed an increase in expression with thyroid hormone replacement. In contrast, cluster 5 consists of genes showing a significant decrease with both a deficiency of TRH and thyroid hormone replacement. It is important to note that among 10445 probes, the sole gene in the entire genome showing the largest and most consistent decrease in the absence of TRH and by supplement with thyroid hormone was the TSHβ gene showing 63% expression of the wild-type pituitary in the absence of TRH, and a further decrease to 6% of the wild-type (Fig 2B, C, and D). These findings demonstrated that TRH is a highly specific regulator of the TSHβ gene in the entire genome. Indeed, genes for other anterior-pituitary hormones such as POMC, FSH, LH, GH, and prolactin, and genes reported to regulate expression of the TSHβ gene including Pit1 and GATA2 were found to show no significant change (Fig. 2C and D).

Other genes classified into cluster 5 were the NR4A1, Egr-1, Gtl2 and Tmsb10 genes as shown in Fig. 2B. The Gtl2 gene is an imprinted gene which is maternally expressed and appears to function as an RNA molecule; multiple splice variants are observed in the available sequence data, and a pituitary transcript variant has been reported to inhibit cell proliferation [41]. The tymosin-beta 10 gene (Tmsb10) is related to cell growth through proliferation, and its expression is associated with brain development in rats and humans. In addition, previous reports indicated enhanced Tmsb10 expression in a variety of human tumors such as renal and thyroid medullary carcinomas and melanomas [42], [43]. Although these two genes were found in the same cluster, cluster 5 to which the TSHβ gene belonged, the signals slightly increased in the absence of TRH, suggesting that these two genes were expressed in a TRH-independent manner. The other two genes, NR4A1 and Egr-1, showed a profile of change similar to that of the TSHβ gene. The NR4A1 mRNA signal in the TRH-deficient pituitary decreased to 70% of that of wild-type mice, and further decreased to 52% of the wild-type level in euthyroid TRH-deficient pituitary (TRHKO+T4). Similarly, the Egr-1 signal in the TRH-deficient pituitary was decreased to 39% of the wild-type value, and reduced to 40% of the wild-type value in the euthyroid TRH-deficient pituitary (TRHKO +T4). Therefore, the reduction of these two genes in TRHKO mice was not recovered by thyroid hormone replacement, indicating that the expression of these two genes was TRH-dependent and probably up-regulated by TRH.

The NR4A1 and Egr-1 genes are known as immediate-early response genes. Egr1- knockout mice have been reported to have LHβ deficiency [44] and impaired liver regeneration [45] but normal thyroid function and intact thyrotrophs in the pituitary, therefore we excluded Egr-1 from the study [46]. In contrast, NR4A1 is known to be an important signal for both the hypothalamo-pituitary-adrenal axis, particularly the pituitary POMC gene and the adrenal Cyp11β2 and HSD3β genes, and the hypothalamo-pituitary-gonadal axis including the 20α–HSD gene in the ovary and the Cyp17 and HSD3β genes in the testis [28]–[35], however, its role in the hypothalamo-pituitary-thyroid axis (HPT axis) remains unclear. We therefore examined the importance of NR4A1 in the HPA axis in subsequent experiments.

Furthermore, the previous analysis of NR4A1 knockout mice demonstrated normal functioning of the adrenal grand, suggesting other homologous factors such as NR4A2 and NR4A3 to compensate for the function of NR4A1 [47]. However, in the present microarray analysis, NR4A2 mRNA expression was not significantly changed and NR4A3 mRNA was not found in the pituitary (Fig. 2D), suggesting that no such compensation in the TRH-deficient mice. Furthermore, there was no report regarding the H-P-T axis and thyroid function in either NR4A1 knockout mice or NR4A1/NR4A3 double knockout mice [47]–[49].

### NR4A1 mRNA Level was Regulated by TRH In vivo

To confirm the results of the microarray analysis, we measured NR4A1 mRNA levels in the pituitary gland by real-time PCR with the TaqMan probe of NR4A1 (Fig 2E). We first confirmed that the NR4A1 mRNA level in the TRH deficient-pituitary was decreased to about 25% of the wild-type level (1.2±0.1 vs. 3.8±0.2, NR4A1 mRNA/GAPDH mRNA, n = 3, *p*<0.01). When the TRHKO were treated with thyroid hormone to be euthyroid (1.5 μg T4/100 g body weight, injected subcutaneously for 14 days), the level of NR4A1 mRNA was not significantly changed (1.5±0.1, n = 3). However, when TRH was given to TRHKO mice by Alzet pump subcutaneously for 7 days, the NR4A1mRNA level completely reverted to normal (2.8±0.4, n = 3) (Fig. 2E), suggesting the expression of NR4A1 mRNA to be TRH-dependent but thyroid hormone-independent. Furthermore, the NR4A2 mRNA level did not differ among the wild-type (0.9±0.1, n = 3), TRHKO (1.0±0.4, n = 3), and TRHKO +T4 (0.8±0.1, n = 3), and NR4A3 mRNA was not detected in the pituitary showing the Ct value of the real-time PCR as 29.5±0.9, while those of NR4A1 and NR4A2mRNA were 24.4±1.65, 27.3±1.0, respectively, suggesting again that compensation of the role of NR4A1 by NR4A2 and 3 might not occur in the anterior pituitary.

### Expression of NR4A1 in the Thyrotrophs and Regulation by TRH In vivo

**Figure 3:**
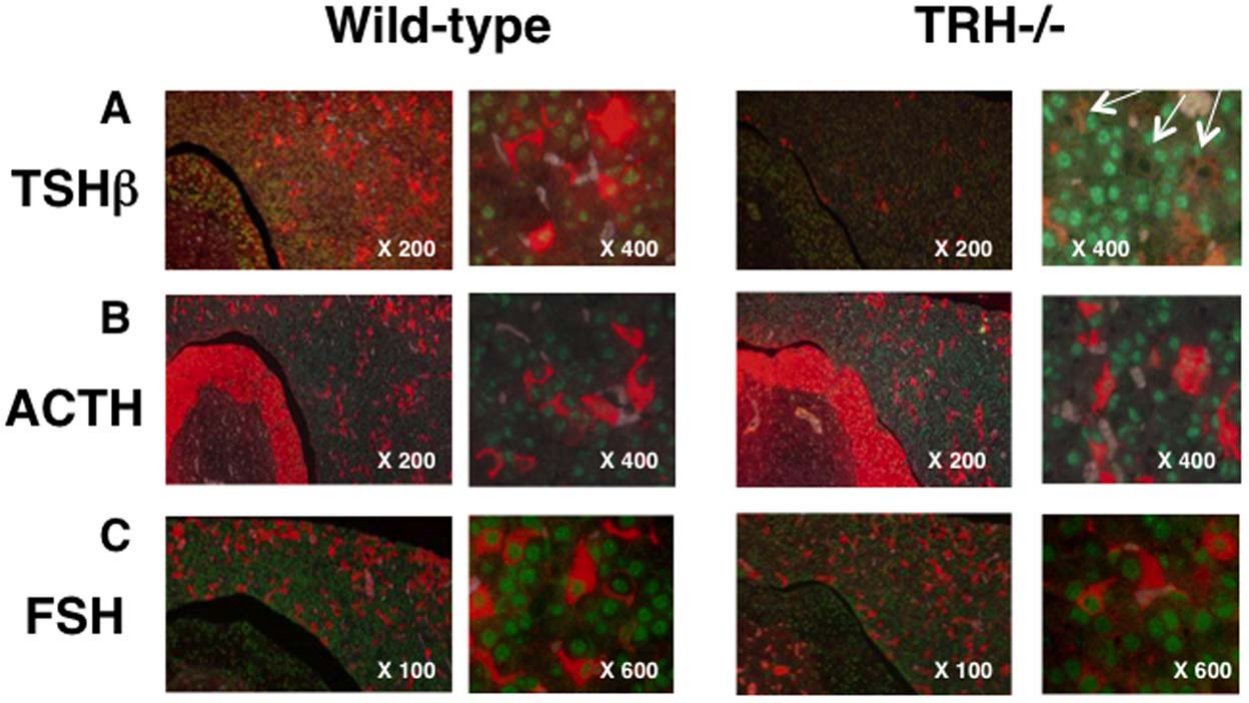
Expression and regulation of NR4A1 by TRH in pituitary thyrotrophs in vivo. The expression of NR4A1 in pituitary thyrotrophs was confirmed by double fluorescent immunohistochemistry. In numbers of cells in the anterior pituitary NR4A1 were expressed and stained as green signals in the nucleus. A) Numbers of cells expressing NR4A1 were also stained with antibody against TSHβ (red signals) in the wild-type pituitary (left panel). As shown in the right panel, the intensity and number of the TSHβ-immunopositive cells were remarkably decreased in the TRH-deficient pituitary, and at X400, most of the NR4A1 expression was reduced or lost in these cells as seen as black dots (indicated by white arrows). B) As observed in Fig 4B and C, the expression of ACTH (B) and FSH (C) was also observed as red signals in the cytoplasm in the anterior lobe. As expected, these ACTH- and FSH- positive cells expressed NR4A1, but the staining intensity was not altered in the TRH-deficient pituitary (right panel).

We next investigated *in vivo* expression and regulation by TRH of NR4A1 in the pituitary thyrotrophs using double-fluorescent immunohistochemistry. As shown in the left panel of Fig 3A, in the wild-type pituitary, NR4A1 shown with green signals was observed in the nucleus of most cells in the anterior, intermediate and posterior lobes. We found in numbers of cells, the coexistence of NR4A1 with TSHβ immunoreactivity shown by red signals in the cytoplasm of the anterior lobe. In contrast, in TRHKO pituitary shown in the right panel, a significant decrease in the staining of TSHβ was observed. However, careful observation indicated that in the remaining weakly-stained cells, NR4A1 expression was absent as shown by a black hole, suggesting the suppressed expression of NR4A1 in the nucleus of the thyrotrophs to be due to the absence of TRH.

**Figure 4:**
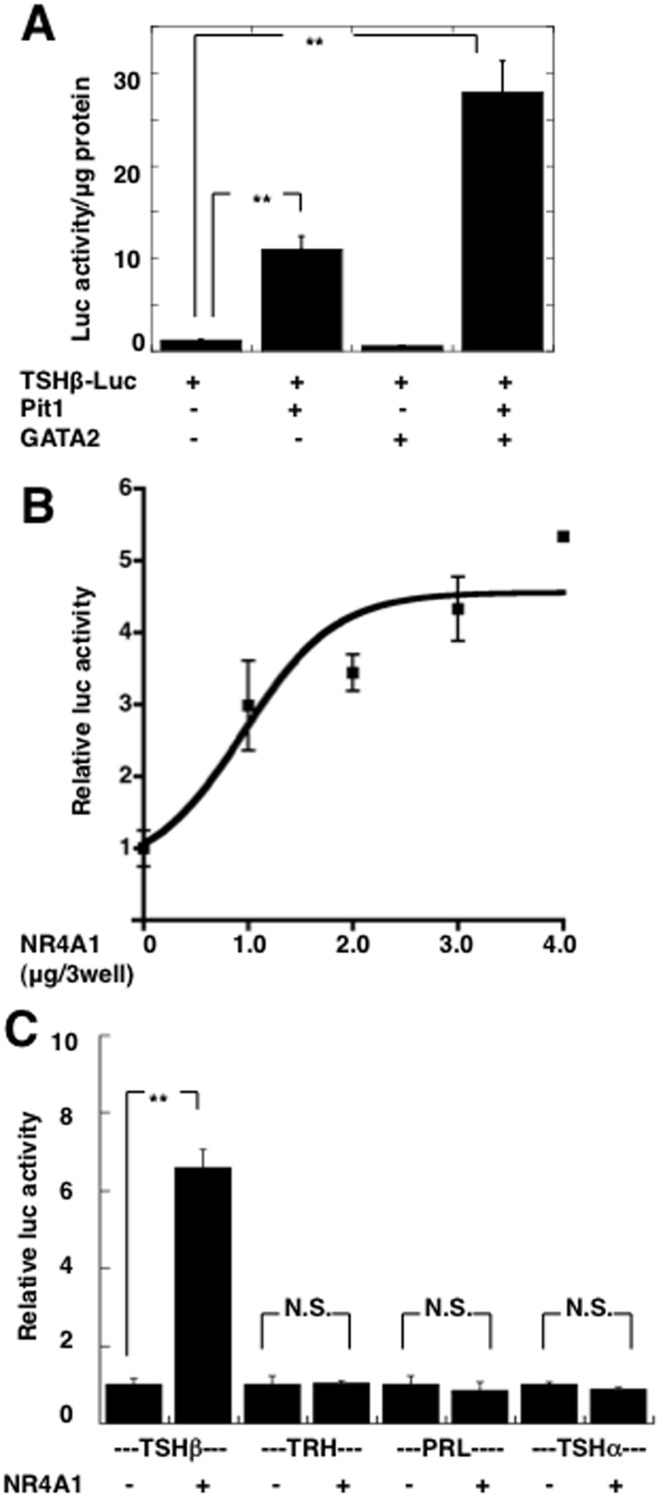
Stimulation of the promoter activity of the TSHβ gene by NR4A1 in vitro. A) The human TSHβ promoter region between bp –1192 and +37 linked to a luciferase reporter gene (pA3TSHβ(−1192∼+37)-Luc) was transfected into CV-1 cells. Expression of Pit1 and GATA2 was necessary for the basal promoter activity of the TSHβ gene. When an expression vector for Pit1 was co-transfected, the promoter activity of the TSHβ was stimulated by 10-fold, but co-transfection with GATA2 did not significantly alter it. However, synergistic stimulation of the promoter activity was observed when Pit1 and GATA2 were simultaneously expressed. B) Under the above conditions expressing Pit1 and GATA2, the TSHβ promoter activity was stimulated by NR4A1 in a dose-dependent manner. When 1 μg of expression vector of NR4A1/3 wells was co-trasfected, the promoter activity was stimulated about 2-fold, and when 3 μg, it reached a plateau at about 6-fold. Relative luciferase activity against the value without expression of NR4A1 is shown. C) Stimulation of the promoter activity by NR4A1 was specific for the TSHβ gene (TSHB). The promoter activities of the thyrotropin-releasing hormone (TRH), TSHα, and prolactin (PRL) genes was not stimulated by overexpression of NR4A1. Relative luciferase activity against the value without expression of NR4A1 in each promoter is shown. Values are represented as the mean ± SEM from at least three experiments. **, *p*<0.01; N.S., not significant.

We also identified ACTH- and FSH- producing cells using specific antibodies in the anterior pituitary. As shown in Fig. 3B and C, many ACTH- and FSH- producing cells expressed NR4A1 in the anterior pituitary. However, the expression of NR4A1 in the ACTH- and FSH- producing cells was not changed by the absence of TRH as shown in the right panel of Fig. 3B and C, suggesting that NR4A1 in the ACTH- or FSH- producing cells was not regulated by TRH *in vivo*.

### Activation of the TSHβ Promoter by NR4A1

We next examined whether NR4A1 stimulates the activity of the TSHβ promoter *in vitro*. As previously reported by us and others, for the full expression of the TSHβ promoter Pit1 and GATA2 were required in CV-1 cells (Fig. 4A) [17], [50]. As shown in Fig 4B, in the presence of Pit1 and GATA2, overexpression of NR4A1 significantly increased the TSHβ promoter activity in a dose-dependent manner and when 4.0 μg of expression vector per plate was used, the activity of the promoter was stimulated by 6-fold the basal level. The effect of NR4A1 could be observed only on the TSHβ promoter, not the TRH promoter or prolactin promoter, suggesting that the effect was specific for the TSHβ promoter (Fig. 4C). Furthermore, the activity of the TSHα promoter was also not stimulated by overexpression of NR4A1. It may be due to the small effect of the absence of TRH on the TSHα mRNA level as compared to the TSHβ mRNA level as shown in Fig. 1C.

**Figure 5:**
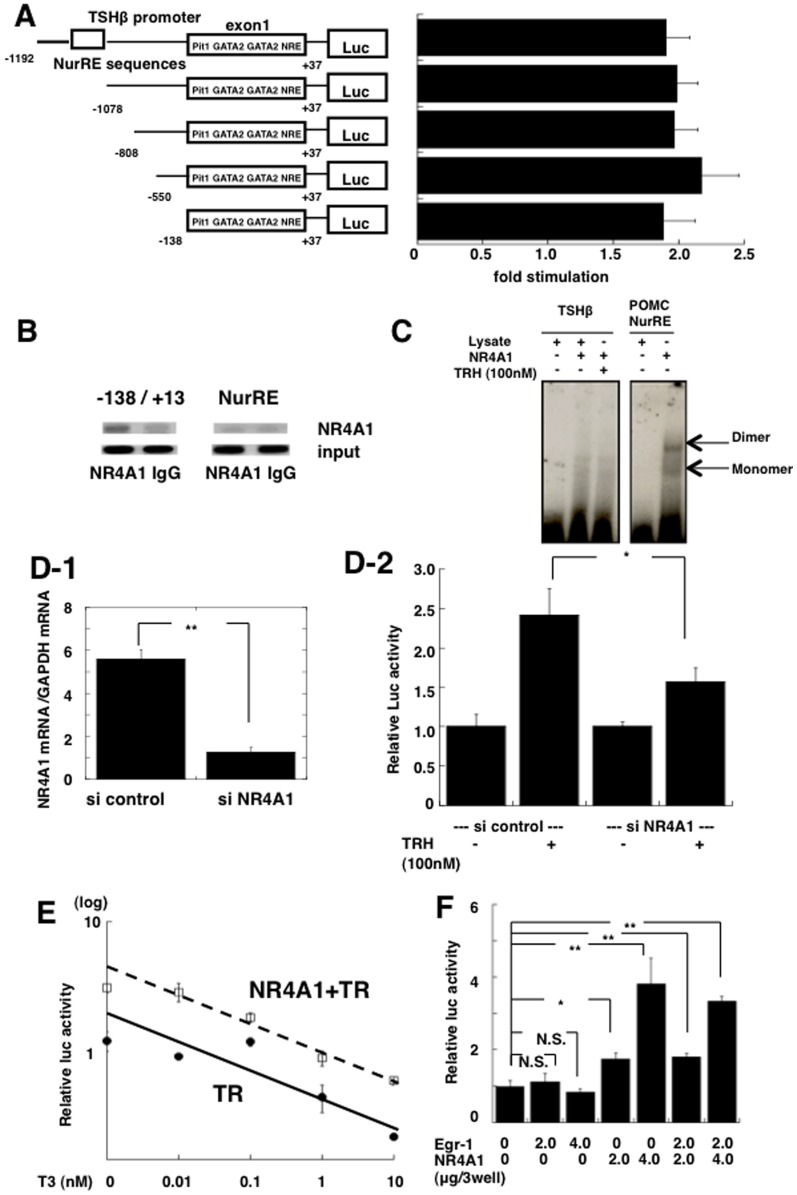
Characterization of NR4A1-induced stimulation of the promoter activity of the TSHβ **gene.** A) To determine the region responsible for the NR4A1-induced stimulation of the TSHβ promoter activity, a series of deletion constructs of the human TSHβ promoter were established. The consensus sequence of the NR4A1 response element (NurRE) was identified in the region between -1091 and -1083 from the transcription start site. The right figure shows the fold-stimulation by NR4A in each deletion mutant of the TSHβ gene. Putative Pit1 and GATA2 binding sites, and the negative thyroid hormone response element (NRE) were indicated as pit1, GATA2 and NRE. All the deletion constructs including pA3TSHβ (−138 ∼ +37) -Luc showed similar stimulation by NR4A1, suggesting the region responsible to be within this area. Fold-increase in activity against that without TRH is shown. B) Chromatin-immunoprecipitation (ChIP) assays were performed with anti-NR4A1 antibody (NR4A1) and normal mouse IgG as a negative control (IgG). GH4C1 cells were transfected with pA3TSHβ (-1192 ∼ +37)-Luc in the presence or absence of 100 n M TRH. Amplified PCR products were stained with ethidium bromide in 2% agarose gels and scanned with a Molecular Imager FX. Chip assays demonstrated that NR4A1 was recruited to the region between -138 and +13 of the TSHβ promoter (-138/+13), but not the region containing NurRE (NuRE). Addition of TRH did not alter recruitment of NR4A1 on the gene (data not shown). All ChIP assays were repeated at least three times. C) The EMSA was performed using a fragment of the radiolabeled POMC promoter containing typical NurRE and a fragment containing the human TSHβ bp -123∼-87. There was no binding of NR4A1 to the TSHβ gene in the absence (−) or presence (+) of 100 nM TRH, while the POMC promoter fragment bound to NR4A1. Arrows indicate NR4A1 bound to the POMC promoter as monomers and dimers. NR4A1 was synthesized by the TNT-coupled reticulocyte lysate system (NR4A1). Lysate indicates un-programmed lysate. D) Effect of knockdown of NR4A1 on the TRH-induced stimulation of the promoter activity of the TSHβ gene. D-1. The NR4A1 mRNA levels were measured after transfection with siCONTROL or siNR4A1 in GH4C1 cells. Transfection of siNR4A1 led to an approximately 80% reduction of the mRNA level after 48 hr. Data are presented as the mean ± SEM from three experiments. **, *p*<0.01 D-2. GH4C1 cells were transfected with siNR4A1 or siCONTROL, and then TSHβ (-1192 ∼ +37)-Luc reporter, Pit1, and GATA2 expression vectors were cotransfected. After incubation with 100 nM TRH for 24 hr, the promoter activity was measured. In the vehicle transfected with siCONTROL, incubation with TRH stimulated promoter activity of the TSHβ gene showing 242±33% of that without TRH stimulation. Knockdown of NR4A1 resulted in significant reduction of TRH-induced stimulation (156% of that without TRH stimulation). Data are presented as the mean ± SEM from three experiments. *, *p*<0.05 E) Negative regulation of the TSHβ gene by thyroid hormone was examined in the presence or absence of NR4A1 in CV-1 cells. Expression vector for thyroid hormone receptor β 1 (TRβ1) was also co-transfected. The responsiveness of the TSHβ promoter activity to thyroid hormone (T3) without expression of NR4A1 was significantly reduced as compared to that with expression of NR4A1, suggesting NR4A1 increased the basal promoter activity and responsiveness to T3 of the TSHβ promoter. F) Effect of NR4A1 and Egr-1 on the promoter activity of the TSHβ gene. Egr-1 affected neither the basal promoter activity of the TSHβ gene nor the NR4A1-induced stimulation of the gene. The amount of vector/3 wells transfected is indicated below the graph. **, *p*<0.01; *, *p*<0.05; N.S. not significant.

In order to determine the region responsible for the effect of NR4A1 on the TSHβ promoter, we first searched for an NR4A1 response element (NurRE)(AAAT(G/A)(C/T)CA) in the TSHβ promoter and found a complete consensus sequence of NurRE (5′-aaatatca-3′) in the region between bp -1091 and -1083 [33], [51]. However, as shown in Fig 5A, transient transfection analysis showed that the deletion of this region of the TSHβ promoter (-1078 ∼ +37 construct) caused a similar response to NR4A1, indicating that this element did not act as a NurRE in the TSHβ gene. Furthermore, a series of deletion mutants including the construct containing the region between -138 and +37 showed a significant response to NR4A1, suggesting that the region between -138 and +37 included the area responsible for the NR4A1-induced stimulation of the TSHβ gene. It has been reported by several investigators that Pit1 and GATA2 bound to this region of the TSHβ gene, and this region is also responsible for the TRH-induced stimulation (Fig. 5A) [17], [52], [53].

### NR4A1 was Recruited to the Region between −138 and +13

There was no consensus sequence for NurRE in the region between bp -138 and +37 of the TSHβ gene, and the EMSA study showed no binding of NR4A1 in the region −123∼−87 of the TSHβ gene, which has been reported to bind to Pit1 and GATA2 and be responsible for TRH-induced stimulation, while the POMC promoter fragment bound to NR4A1 as both dimers and monomers (Fig. 5C). Therefore, we expected indirect recruitment, and examined whether NR4A1 was recruited to this region by a chromatin-immunoprecipitation (ChIP) assay and a specific antibody against NR4A1. The ChIP assay demonstrated that NR4A1 appeared to be recruited both in the presence or absence of TRH, while the region containing a NurRE sequence (between -1091 and -1083) did not recruit NR4A1 (Fig. 5B).

### Knockdown of NR4A1 by siRNA Reduced TRH-induced Stimulation of the TSHβ Gene

In order to examine the importance of NR4A1 for activation of the TSHβ promoter by TRH, we next knocked-down NR4A1 by siRNA and examined the effect of NR4A1 on the gene. Treatment with siRNA (siNR4A1) induced a reduction of NR4A1 mRNA to 20% of the control siRNA (siControl) in a rat pituitary cell line, GH4C1 cells (Fig. 5D-1). The level of TSHβ promoter activity induced by TRH was 2.5-fold high in the control cells transfected with siControl, while activation by TRH was significantly suppressed to about 40% of that of siControl when NR4A1 expression was knocked down (Fig. 5D-2), suggesting the importance of NR4A1 for TRH-induced stimulation of TSHβ promoter activity.

### Expression of NR4A1 Shifted the Dose-response Curve of the TSHβ Gene Against Thyroid Hormone

When compared to the curve of dose-response repression of the TSHβ gene by thyroid hormone in the presence or absence of overexpression of NR4A1 in CV-1 cells, the curve was shifted to the right in the presence of NR4A1, indicating that NR4A1 increased the responsiveness of the TSHβ gene to thyroid hormone *in vitro* (Fig 5E). This phenomenon was similar to that of the *in vivo* experiment comparing TSHβ mRNA levels between the wild-type mice and TRHKO differing thyroid hormone status shown in Fig. 1D.

Furthermore, another typical early response gene, Egr-1, did not alter the promoter activity of the TSHβ gene in the presence or absence of NR4A1 (Fig 5F).

### Immediate-early Response of NR4A1 mRNA via TRH through the PKC and MAPK Pathways

**Figure 6:**
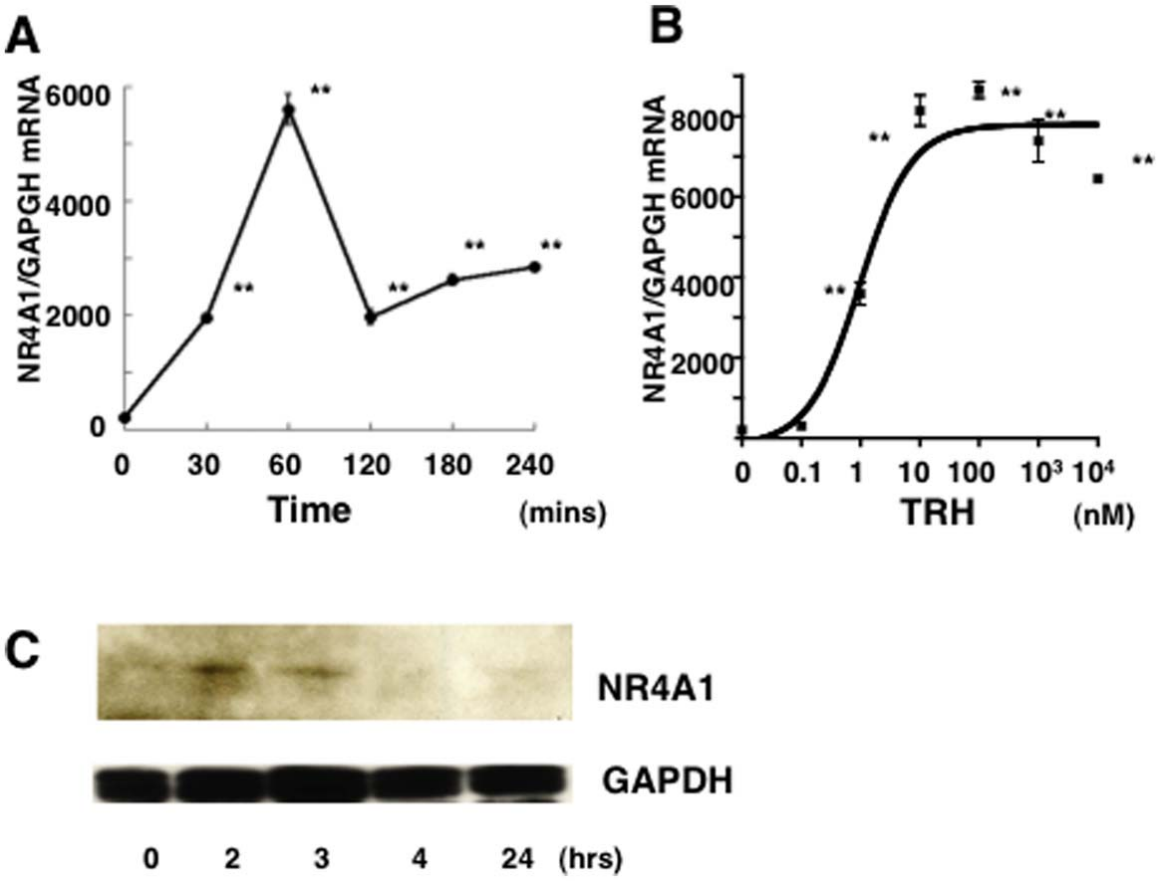
Immediate-early response of NR4A1 mRNA and protein levels to TRH in GH4C1 cells. A) Incubation with 1μM TRH led to a remarkable increase of the endogenous NR4A1 mRNA level with a peak within 1 hr (51-fold) and then an immediate reduction in 120 mins in GH4C1 cells. B) The TRH-induced increase of the NR4A1 mRNA level was observed in a dose-dependent manner. The minimum effect was observed with 0.1 nM TRH and then reached a plateau at 10 nM. C) Western blot analysis in GH4C1 cells demonstrated that incubation with 100 nM TRH led to a significant increase of NR4A1 protein (NR4A1) within 2 h, and NR4A1 was then degradated in 4 h, while the level of GAPDH was not altered.

We next examined how TRH stimulates the expression of endogenous NR4A1mRNA in GH4C1 cells. Incubation with 100 nM TRH induced a marked and rapid increase in NR4A1 mRNA levels with a peak at 60 mins that was 51-fold the basal level, and then a rapid reduction to 19% of the basal level within 120 mins (Fig 6A). Furthermore, treatment with TRH increased NR4A1 mRNA levels in a dose-dependent manner with a minimum dose of 0.1 nM and a plateau at 10 nM (Fig. 6B), suggesting the NR4A1 mRNA to act as an immediate-early response gene for TRH-stimulation. Furthermore, Western blot analysis of GH4C1 cells demonstrated that incubation with 100 nM TRH increased NR4A1 expression at the protein level within 2 h with a peak at 2 h, and then a decrease in 4 h (Fig 6C). In addition, TRH did not alter the pit1 mRNA level, and GATA2 and POMC mRNAs were not detected in GH4C1 cells (data not shown).

**Figure 7:**
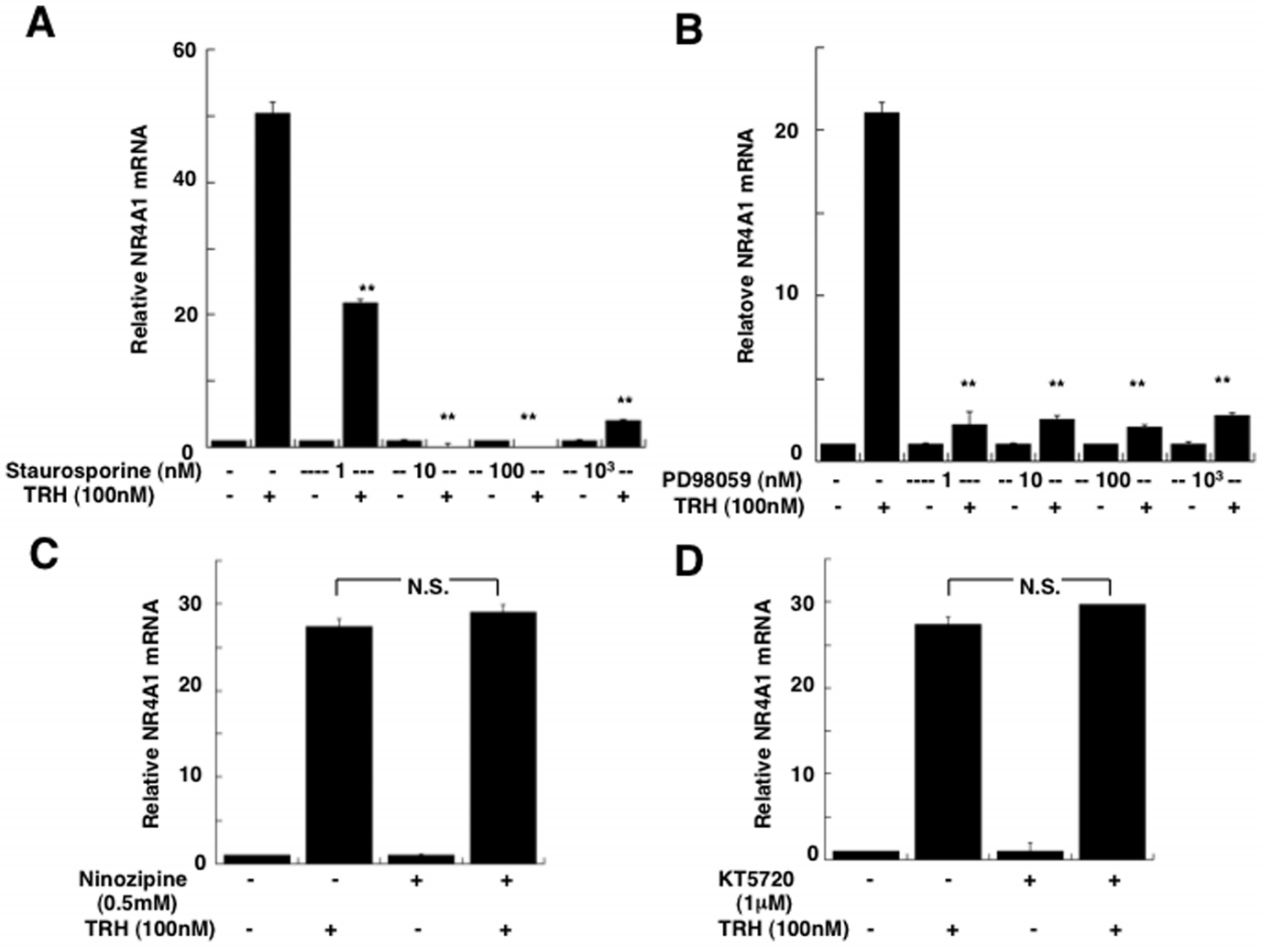
Regulation of NR4A1 mRNA by TRH through the PKC and MAPK pathways. The signal transduction pathways for the TRH-induced stimulation of NR4A1 mRNA expression were determined using several inhibitors in GH4C1 cells. After overnight incubation in DMEM without serum, GH4C1 cells were treated for 1 hr with a PKC inhibitor, Staurosporine (A), a MAPK inhibitor, PD98059 (B), a Ca channel inhibitor, nimozipine (C), and a PKA inhibitor, KT5720 (D). Significant inhibition of 100 nM TRH-induced stimulation of endogenous NR4A1 mRNA expression was observed with 1.0∼ nM Staurosporine and 1.0 ∼ nM PD98059, but not 0.5 nM nimozipine or 1 μM KT5720. The value relative to that without TRH is shown. Data are presented as the mean ± SEM. from three experiments. **, *p<*0.01.

To explore which signal transduction pathway was involved with TRH-induced NR4A1 mRNA expression, we used several specific inhibitors. As shown in Fig. 7A, treatment with 100 nM TRH significantly stimulated the expression of endogenous NR4A1 mRNA in 60 mins, however, an prior incubation with a inhibitor for PKC (1 nM), staurosporine significantly inhibited this stimulation by 60%, and 10 nM staurosporine showed about 90% inhibition. Similarly, an inhibitor for MAPK, PD98059 (1–1000 nM), showed significant inhibition of TRH-induced stimulation of NR4A1 mRNA (90% of the control)(Fig. 7B). In contrast, an inhibitor for Ca2+ channels, nimodipine, or for PKA, KT5720, did not affect it (Fig. 7C and D). These findings suggested that TRH-induced NR4A1 mRNA expression was mediated through both PKC and MAPK pathways.

### Stimulation of the NR4A1 Promoter Activity by TRH through TRH Receptors

**Figure 8:**
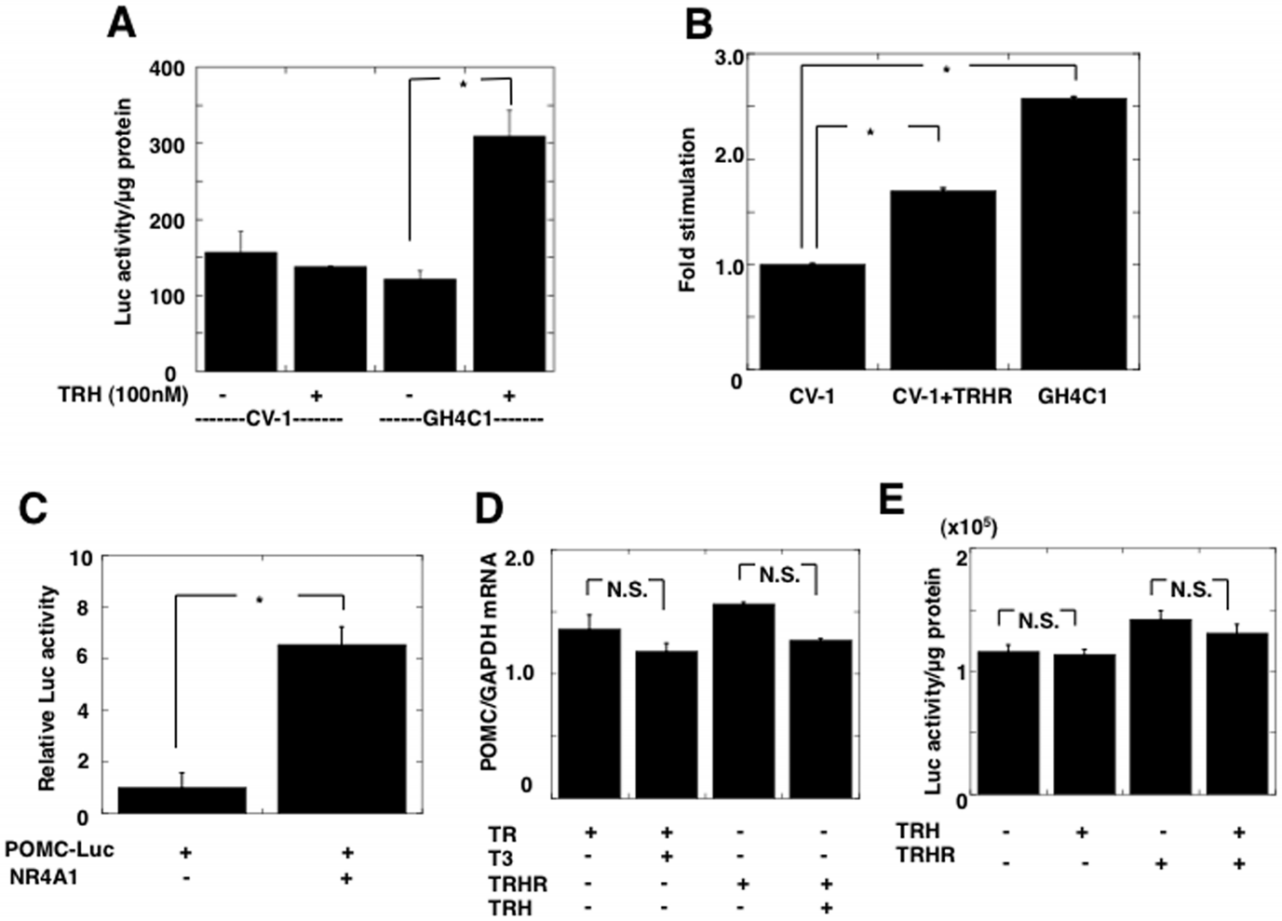
TRH stimulates the promoter activity of the NR4A1 gene through TRH receptors. A) A fragment spanning bp −1295 and +152 of the promoter of the human NR4A1 gene fused to a luciferase reporter gene (pA3NR4A1(−1295∼+152)-Luc) was transfected into CV-1 cells (CV-1) that express no endogenous TRH receptors (TRHRs) and GH4C1 cells (GH4C1) expressing endogenous TRHRs. In CV-1 cells, 100 nM TRH did not stimulate the promoter activity of the NR4A1 gene, in contrast, it led to a significant increase in GH4C1 cells (255±33% of the control without TRH, n = 3, *p*<0.05). B) When TRH receptors were expressed in CV-1 cells (CV-1+TRHR), 10^−7^ M TRH induced a significant increase of NR4A1 promoter activity, suggesting that TRH exhibited stimulation of the NR4A1 gene through TRH receptors. Fold-increase on incubation with 100 nM TRH is represented. The value in GH4C1 cells is also shown as a control. C) The rat POMC promoter containing the region from bp −706 to +64 fused to a luciferase reporter (POMC-Luc) was transfected into At-T20 cells expressing the endogenous POMC gene. The promoter activity was significantly increased about 6-fold by overexpression of NR4A1. D) The level of endogenous POMC mRNA was not altered either by incubation with expression of TR and T3 or by incubation with expression of TRH receptors (TRHRs) and 100 nM TRH in At-T20 cells. E) The POMC promoter activity was not stimulated by incubation with 100 nM TRH even with expression of TRH receptors (TRHRs), suggesting that the effect of TRH occurred in a promoter-specific manner. Data are presented as the mean ± SEM. from three experiments. *, *p*<0.05; N.S., not significant.

To examine whether TRH stimulates transcription of the NR4A1 gene, we performed a transient transfection assay with a NR4A1 promoter in GH4C1 cells expressing endogenous TRH receptors and CV-1 cells without the expression. In GH4C1 cells, incubation with 100 nM TRH induced a significant stimulation of the NR4A1 promoter activity (257±22% of the control. n = 3, *p<*0.05). In contrast, no significant stimulation was observed in CV-1 cells (Fig. 8A). To determine whether TRH-induced NR4A1 mRNA expression is mediated through TRH receptors, we performed a similar experiment in CV-1 cells with and without expression of TRH receptors. As shown in Fig 8B, incubation with 100 nM TRH significantly stimulated the activity of the NR4A1 promoter with expression of TRH receptors, but, no change was observed without, suggesting the effect of TRH to be mediated through its receptors. Conversely, the POMC promoter activity was significantly stimulated by overexpression of NR4A1 to about 6-fold the basal level in At-T20 cells expressing endogenously POMC gene (Fig. 8C). However, incubation with TRH did not alter endogenous the POMC mRNA levels in At-T20 cells with and without TRH receptors (Fig. 8D). In addition, the POMC promoter was not affected by incubation with TRH in the presence or absence of TRH receptors (Fig. 8E). These findings suggested that the effect of TRH on NR4A1 mRNA and subsequent stimulation of the TSHβ promoter occurred in a receptor- and promoter-specific manner.

## Discussion

In the present study, we first demonstrated that TRH altered the basal expression of the TSHβ gene and the responsiveness of the gene to thyroid hormone *in vivo* by using TRHKO differing in thyroid hormone status. When TRH-deficient mice were supplemented with thyroid hormone, the elevated serum TSH level became normal. In contrast, the decreased TSH mRNA levels in the TRH-deficient pituitary were further decreased, and this reduction was found predominantly in the TSHβ subunit. Although thyroidectomy caused severe hypothyroidism in TRH-deficient mice, the increases in serum TSH levels and pituitary TSHβ mRNA levels were insufficient. Analysis of the correlation between the serum thyroid hormone level and the corresponding serum TSH and TSHβ mRNA levels clearly demonstrated that TRH altered the basal activity and responsiveness to thyroid hormone of the gene. In addition, the serum TSH level was more profoundly affected in animals with severe hypothyroidism in the absence of TRH. These findings demonstrate that TRH is a major up-regulator of TSH synthesis, altering the basal activity and the responsiveness to thyroid hormone of the gene, and TRH also affected TSH production by post-transcriptional mechanisms *in vivo*.

In hypothyroid status TRH and thyroid hormone receptors (TRs) both may be required for hypothalamic-pituitary-thyroid axis for stimulation of the production and secretion of TSH *in vivo* [54]. Unliganded thyroid hormone receptors exert a silencing effect in genes regulated positively by thyroid hormone but, we and others reported that unliganded TRs have a constitutively stimulating effect on the genes regulated negatively by thyroid hormone *in vitro*, such as the TSH gene [55]–[58]. Therefore, one may expect that unliganded TRs function as an up-regulator in hypothyroid animals. However, when animals who possessed no TRs (TRα1−/−β−/−) were rendered to hypothyroidism, their serum TSH and TSHβ mRNA levels in the pituitary were equivalent to those in the wild-type mice, demonstrating that TRs are not required and not involved with the up-regulation of the TSH gene in the hypothyroid status [59]. Furthermore, we have recently reported the predominant action of TRH compared to TRs in the hypothalamic-pituitary-thyroid axis using TRβ/TRH double knockout mice (KO) [39]. TRβKO mice had an impairment of negative feedback regulation by thyroid hormone, resulting in a significant rise in serum T4 levels and showed resistance to thyroid hormone with elevated serum TSH levels compared with wild-type mice [39], [60]–[62]. Double KO showed a reduced serum thyroid hormone level compared with simple TRβKO and showed only a slight increase in TSH levels compared with wild-type mice. In addition, when TRβKO and double KO were rendered to severe hypothyroid by treatment with PTU, double KO failed to show a significant rise in serum TSH levels as compared to TRβKO. Therefore, TRH is essential for the stimulation of the TSHβ gene under both euthyroid and hypothyroid conditions.

How does TRH up-regulate TSHβ gene expression *in vivo*? In the present study, the cDNA microarray and K-means cluster analysis with TRH-deficient pituitary revealed that among whole genes a gene in which most dramatic effect of TRH was observed is the TSHβ gene, and no changes were observed in other pituitary hormones including GH, POMC, PRL, FSH, or LH. In addition, NR4A1 was a gene showing TRH-dependent expression and showed a similar profile to the TSHβ gene. However, expression of Pit1 and GATA2, other candidates for a factor related to TRH-induced stimulation of the TSHβ gene, was not altered by TRH or thyroid hormone replacement *in vivo*. Therefore, we focused on the effect of NR4A1 on the transcription of the TSHβ gene *in vitro.* Overexpression of NR4A1 clearly stimulated the promoter activity of the TSHβ gene in a time- and dose-dependent manner. In addition, we found that incubation with TRH markedly stimulated expression of NR4A1 mRNA by over 50-fold in an early time within 60 minutes. Furthermore, immunocytochemistry studies demonstrated co-expression of NR4A1 with TSHβ in the anterior pituitary, and in addition the expression was significantly decreased in the TRH-knockout mice. To our knowledge, this study is the first demonstration of the presence of NR4A1 even in ACTH- and FSH- producing cells by immunohistochemistry [63]. Taken together, we demonstrated that NR4A1 is an up-regulator working down-stream of TRH in the anterior pituitary *in vivo.*

**Figure 9:**
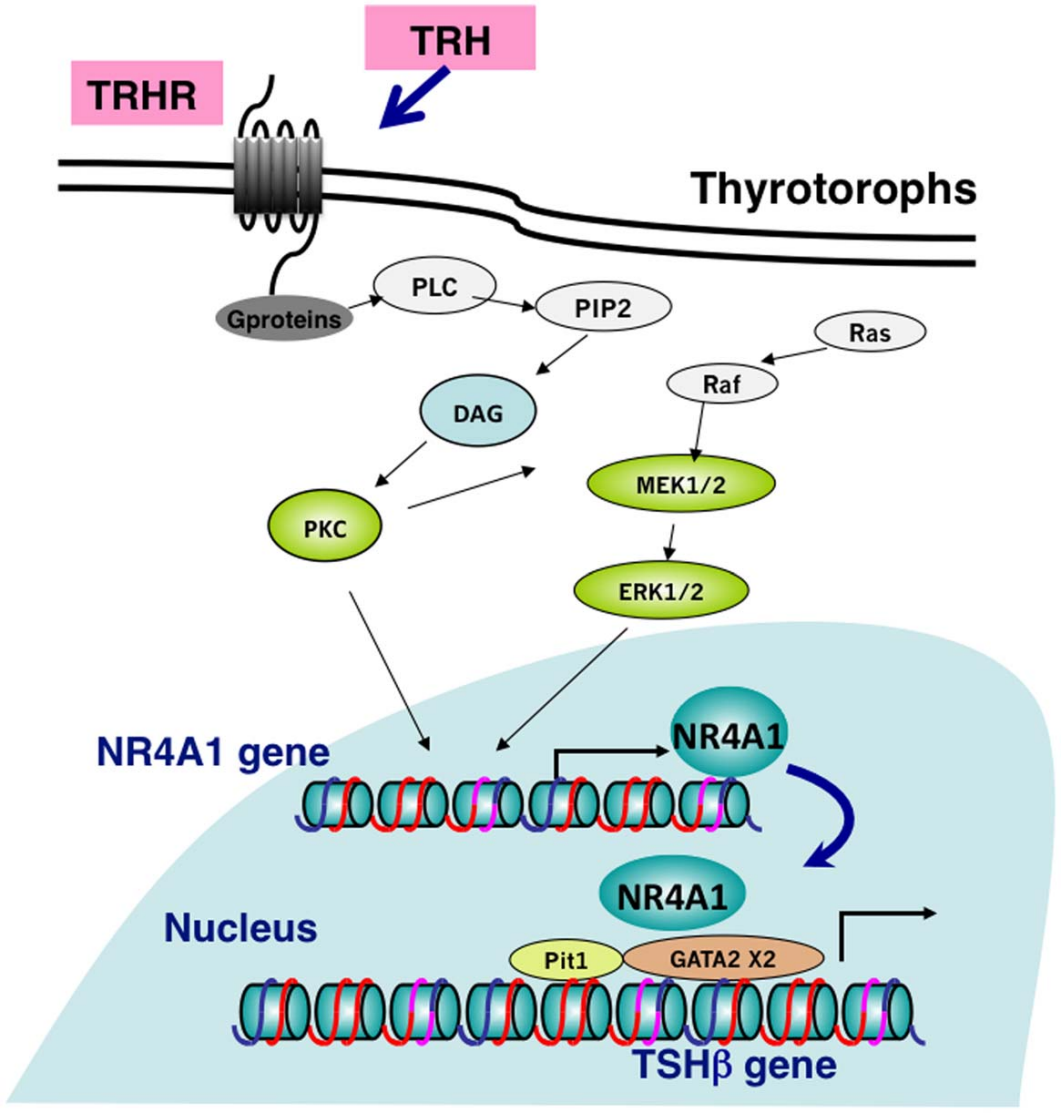
Proposed models of the TRH-NR4A1-TSHβ pathway. TRH stimulates the transcription of the NR4A1 gene through TRH receptors, and PKC and MAPK pathways within 1 hr. The increased amount of NR4A1 stimulates TSHβ promoter activity, which may act cooperatively with Pit1 and GATA2 on the gene.

The analysis with a series of deletions of the promoter region of the TSHβ gene demonstrated that the responsible region of TRH-NR4A1 induced-stimulation of the TSHβ gene was between bp -138 bp and +37 from the transcription start site (TSS). Furthermore, although no consensus sequence for binding site for NR4A1 was found in this region, Chip assay with a specific antibody against NR4A1 revealed that NR4A1 was recruited to this region. Steinfelder et al, also demonstrated with a transient transfection study and deletion analysis of the human TSHβ gene that the region between -128 and +8 in relation to TSS is responsible for TRH-induced stimulation of the gene [16]. This region was close to the TSS and contained binding sites for Pit1 and GATA2, and the thyroid hormone inhibitory element (nTRE). Steinfelder et al. reported that the response element of the TSHβ gene to TRH was located in two discrete regions (-128 to -92 base pairs and -28 and +8 base pairs). The upstream site contains a DNA sequence with homology to the DNA-binding site for a pituitary specific transcription factor, Pit1, and the downstream site overlaps with the thyroid hormone inhibitory element [16], [17]. Furthermore, a recent study of pituitary-specific GATA2 Knockout mice demonstrated reduced pituitary TSH content and serum TSH levels, and pituitary TSHβ mRNA showed a defective response to hypothyroidism [64]. Two regions of the mouse TSHβ gene have a sequence homologous to a consensus motif of the GATA binding site, AGATGC, at the region between -110 and -105 to the TSS, and AGATAA between -98 and -93 [17], and these sequences are perfectly conserved in rodents and humans [20]. Although both Pit1 and GATA2 have been reported to be essential for the expression of the TSHβ gene in thyrotrophs, the present study demonstrated that knockdown of NR4A1 with siRNA significantly reduced TRH-induced stimulation of the promoter activity of the TSHβ gene. Therefore, as shown in Figure 9 as a proposed model of action of TRH on the TSHβ gene, NR4A1 may also be critical and cooperatively work with Pit1 and GATA2 as an up-regulator of TRH-induced stimulation of the TSHβ gene through this region -128 and +8 close to the TSS. Although interactions of NR4A1 with several transcription factors and coregulators, including silencing mediator for retinoid and thyroid hormones (SMRT), steroid receptor coactivator (SRC-1), TRAP220, and SIN3B/KDM5A and Z3 (ZMYND8-containing) complex have been reported, those with Pit1 and GATA2 remain to be studied [52], [65]–[67].

NR4A1 was originally isolated as a gene induced by nerve growth factor in PC12 cells [23]. Subsequently, it has been reported to act as a stimulus-induced transcriptional activator in many tissues and organs, and recently been implicated in apoptosis, cell cycle regulation, inflammation, carcinogenesis and metabolism [24]–[27]. In addition, NR4A1 plays an important role in the hypothalamo-pituitary-adrenal (HPA) axis [32], [33], [36], [37], [51]. Philips et al. reported that the regulation of transcription of the POMC gene by CRH was mediated through activation of NR4A [33]. However, NR4A1-deficient mice showed normal adrenocortical function, but subsequent studies demonstrated that other members of a subfamily of nuclear receptors such as NR4A2 (Nurr1) and NR4A3 (Nor-1) might compensate for a loss of NR4A1 and maintain sufficiently normal steroidogenesis *in vivo* [37], [47]. We also examined expression of NR4A2 and NR4A3 mRNA in GH4C1 cells, however, neither significant expression nor effect of TRH was observed on these mRNA levels. In addition, as mentioned above knockdown of NR4A1 in GH4C1 cells significantly reduced TRH-induced stimulation of the TSHβ gene. Therefore, NR4A1 appeared to be essential for thyrotrophs in the pituitary gland. Furthermore, the pituitary–thyroid–axis in NR4A1-deficient mice has not been characterized, and so will be interesting to study in the future [47], [48].

NR4A1 has also been well characterized as a regulator in the hypothalamo-pituitary-gonadal (HPG) axis. GnRH stimulated NR4A1 expression in pituitary gonadotropes and stimulated transcription of the LH gene [68], [69]. In addition, in the testis and ovary, expression of the steroidogenic acute regulatory (StAR) gene and steroidogenesis are regulated by the LH-NR4A1 pathway [70]–[72]. Thus, NR4A1 would be a common transducer of transcriptional signals of hypothalamic hormones for the regulation of targeted pituitary hormone genes in the anterior pituitary gland.

## Methods

### Reagents and Solutions

Inhibitors (Staurosporine, PD98059, nimodipine and KT5720) were purchased from Sigma–Aldrich (St. Louis, MO). Staurosporine, PD98059 and KT5720 were dissolved in DMSO, and nimodipine in methanol. All vehicles were incubated with the same concentration of dissolved solvent. TRH was kindly provided by Tanabe-Mitsubishi Pharmaceutical Company, Japan.

### Animals

Procedures for animal care and use in this study were approved by the Review Committee on Animal Use at Gunma University, Maebashi, Japan. Animals were maintained on a 12-hour light and 12-hour dark schedule (lights on at 6.00 h) and fed laboratory chow and tap water ad libitum. All experiments were performed between 9.00 and 11.00 h. The mice with thyroxin (T_4_) replacement received 1.5 μg T4/100 g body weight, subcutaneously for 14 days before the experiment. TRH replacement was performed with 1 mg TRH/kg body weight/day by Alzet pump (micro-osmotic pomp model 1002, Alzet, U.S.A.) subcutaneously for 7 days before the experiment.

Experimental hypothyroid mice were generated by thyroidectomy under microscopy. Thyroidectomized mice were given with 1% CaCl2 in drinking water to prevent hypocalcemia due to the simultaneous removal of the parathyroid gland. A mild hypothyroidism was induced by 0.1% MMI in drinking water for 7 days with regular chow that contained iodine.

### Radioimmunoassay (RIA)

Serum TSH levels were measured by a specific mouse TSH RIA with mouse TSH/LH reference (AFP51718MP), mouse TSH antiserum (AFP98991) and rat TSH antigen (NIDDK-rTSH-I-9), all of which were obtained from Dr. A.F. Parlow (Harbor-UCLA Medical Center, Torrance, USA). Serum free T_4_ levels were determined by RIA (GammaCoat Free T4, DiaSorin Inc. USA). All assays were performed in duplicate and had a detection limit of 0.16 ng/dl.

### RNA Extraction and Real-time PCR

Total RNA was prepared from mouse pituitary, GH4C1 cells and At-T20 cells using ISOGEN (Nippongene, Toyama, Japan) according to the manufacturer’s instructions. Then cDNA was reverse-transcribed from 300 ng of total RNA (TaqMan Reverse Transcription Reagents, Applied Biosystems, Tokyo, Japan), and 0.5 μl was subjected to real-time PCR. All reactions were performed in triplicate using TaqMan probes and an Applied Biosystems 7500 sequence detection system. TaqMan probes for NR4A1 (Mm01300401, Rn01533237), NR4A2 (Mm00443060, Rn00570936), NR4A3 (Mm01354011, Rn00569312), Pit1 (Mm00476852, Rn00564562), GATA2 (Mm00492300, Rn00583735), POMC (Mm00435874, Rn00595020) and GAPDH (Mm99999915, Rn99999916) were purchased from Applied Biosystems. The expression level of each mRNA relative to that of GAPDH was calculated using a standard curve, and the relative quantification method was performed as described in ABI User Bulletin #2. All experiments were repeated at least twice.

### cDNA Microarray Analysis and K-means Cluster Analysis

Total RNA was prepared from each mouse pituitary using ISOGEN (Nippongene, Toyama, Japan) according to the manufacturer’s instructions. cRNA was then prepared and hybridized to an Affymetrix Mouse Gene chip 320 2.0 Array. Only genes with raw data greater than 100 signals for all conditions were included in the analysis. Genes after exclusion of absent signals were subjected to a cluster analysis using a K-means algorithm. The hierarchical cluster analysis was carried out using the computer program Genowiz™ (ver. 3.2).

### Double-fluorescent Immunohistochemistry

A rabbit polyclonal antibody for NR4A1 (LS-B114) was purchased from LifeSpan BioSciene (Seattle, WA, USA). Antibodies for mouse TSHβ (M-16: sc-7815) and was purchased from Santa Cruz Biotechnology Inc., (Santa Cruz, CA, USA). An ACTH antibody was purchased from DAKO Corp. (Glostrup, Denmark), and a follicle-stimulating hormone (FSH) antibody (MON5012) was obtained from MONOSAN (MONOSAN, Uden, Netherlands). Fluorescently labeled secondary antibodies used were TSHβ (red), Alexa Fluor 594 donkey anti-goat IgG (Invitrogen, California, USA), NR4A1(green), FITC: polyclonal goat rabbit immunoglobulins (HRP) (DAKO Glostrup Denmark); ACTH (red), Alexa Fluor 594 rabbit anti-mouse IgG (Invitrogen, California, USA), NR4A1(green), FITC: polyclonal goat rabbit immunoglobulins (HRP) (DAKO Glostrup, Denmark); FSH (red, Alexa Fluor 594 donkey anti-mouse-IgG(H+L) (Invitrogen, California, USA), NR4A1 (green), and Alexa Fluor 488 goat anti-rabbit-IgG (H+L) (Invitrogen, California, USA).

Pituitaries were excised and fixed with 4% paraformaldehyde at 4°C overnight. Tissues were embedded in paraffin wax and sectioned sagittally in 4 μm-thick slices. The sections were pretreated with 0.3% hydrogen peroxide for 30 min for the quenching of endogenous peroxidase activity and then autoclaved for 10 min at 120°C. They were rinsed in TBS (Tris-buffer saline) and incubated with skim milk for 30 min at room temperature to block non-specific antibody binding, and treated with the above antibodies (TSHβ, ACTH, or FSH) at 4°C overnight. After another three washes in TBS, the sections were incubated with the secondary antibodies, then washed in TBS, and treated with the NR4A1 antibody at 4°C overnight. After three washes in TBS, the sections were incubated with the secondary antibodies, washed in TBS, and treated with Vectashield mounting medium (VECTOR Laboratories, CA, USA). We observed the slides with a BX52 biological microscope and DP70 digital camera (OLYMPUS, Tokyo, Japan).

### Mammalian Cell Culture

CV-1 (a monkey kidney cell line) [73], GH4C1 (a rat pituitary cell line) [74] and At-T20 (a mouse pituitary adenoma cell line) [75] cells were grown in Dulbecco’s modified Eagle’s medium (DMEM) supplemented with 10% fetal bovine serum (FBS), as described previously [76]. In some experiments, GH4C1 cells were starved in DMEM without serum overnight, then pre-treated with several inhibitors for signal transduction at the indicated concentrations 30 min before the treatment with TRH.

### Plasmid Constructions

Expression vectors for the mouse NR4A1, Egr-1, GATA2, Pit1 and TRH receptor (TRHR) were prepared by PCR and verified by sequencing of the DNA. The mouse NR4A1, Egr-1, GATA2 and Pit1 cDNAs were subcloned into the vector pcDNA^TM^3.1 D/V5-His-TOPO, and TRHR was subcloned into the vector pCDM8 for *in vitro* transcription/translation and transient expression analyses. The human TRβ_1_ cDNAs were subcloned into the vector pKCR_2._ The human NR4A1 promoter containing 1,295 bp of the 5′ flanking sequence and 152 bp of exon 1(pA3NR4A1(-1295∼+152)-Luc), the rat POMC promoter containing 706 bp of the 5′ flanking sequence and 64 bp of exon 1, the human TSHβ-Luc containing 1,192 bp of the 5′ flanking sequence and 37 bp of exon 1 (pA3TSHβ(−1192∼+37)-Luc), the human prolactin gene containing 332 bp of the 5′ flanking sequence and 65 bp of exon 1 (pA3PRL-Luc), and the human TSHα-Luc containing 486 bp of the 5′ flanking sequence and 44 bp of exon 1 (pA3TSHα-Luc) [77] were fused to a firefly luciferase reporter plasmid (pA3Luc).

### Cell Transfection and Luciferase Assay

Twenty-four hours before transfection, cells were split into 6-well plates at subconfluency. The transient transfection was performed using a calcium phosphate precipitation method, as described previously [76]. The total amount of transfected plasmid was adjusted by adding an empty expression vector in all experiments. Sixteen hours after transfection, the medium was changed to DMEM supplemented with 10% FBS treated with AG1-X8 resin (Bio-Rad) and activated charcoal (Sigma) to remove thyroid hormones. Cells were further incubated in the presence or absence of TRH at the indicated concentration.

To determine luciferase activity, cell monolayers were rinsed twice with PBS, then lysed with 300 μl of 25 mM glycylglycine (pH 7.8) containing 15 mM MgSO_4,_ 4 mM EGTA, 1 mM dithiothreitol, and 1% v/v Triton X-100. Cells were scraped from the dishes and centrifuged at 12.000× g for 5 min at 4°C. Assays for Luc activity were performed using 150 μl aliquots of cell lysate and 210 μl of 25 mM glycylglycine (pH 7.8) containing 15 mM MgSO_4,_ 4 mM EGTA, 3.3 mM KPO_4,_ 1 mM dithiothreitol, and 0.45 mM ATP. The reaction was initiated by addition of 200 μl of 0.2 mM d-luciferin and light emission was measured for 10 seconds using a luminometer. Luciferase activity was expressed as arbitrary light units per microgram of cellular protein. All the transfection experiments were repeated at least twice with triplicate determinants.

### Western Blot Analysis

GH_4_C_1_ cell lysates were prepared in RIPA buffer and 100 μg/ml PMSF, 30 μl/ml Aprotinin, and 1 μmol/ml sodium orthovanadate, and then passed through a 23-gauge needle on ice. For cell and tissue extracts, the samples were incubated on ice for 30 min in RIPA buffer. Insoluble cell debris was removed by centrifugation at 10.000 X g for 10 min. Aliquots of protein-containing supernatant were stored at −80°C. Protein concentrations were determined by the Bradford method using the Bio-Rad protein assay reagent (Bio-Rad Laboratories, Inc. Tokyo, Japan). The lysates (40 μg) were resolved by SDS-PAGE gel (10%) and transferred to a polyvinylidene fluoride membrane (Hybond-P, Amersham Biosciences, Tokyo, Japan) with a semidry system (BIO CRAFT, Tokyo, Japan) for detection of NR4A1. The blots were blocked for 1 h with 5% skim milk in Tris-buffered saline with 0.1% Tween 20 (TBST) and probed for 16 h with a primary antibody against NR4A1 (LS-B114, LifeSpan BioScienes, Seattle, WA, USA). After three washes with Tris-buffered saline with 0.1% Tween 20, antigen-antibody complexes were detected using a peroxidase-conjugated secondary rabbit antibody and an enhanced fluoro-chemiluminent system (ECL-plus; Amersham Biosciences).

### Small Interfering RNA (siRNA) Experiments

Pooled siRNA oligonucleotides targeting NR4A1 were designed, synthesized and annealed at Dharmacon Research, Lafayette, CO (siGENOME SMART pool NR4A1, L-100466-01)(siNR4A1). Pooled unrelated siRNA (siCONTROL non-targeting siRNA pool, D-001810-0X)(siControl) was used as a control. These siRNAs were transfected into GH4C1 cells by the lipofection method (Lipofectamine RNAiMAX™, Invitrogen, California, USA). Briefly, in the 6-well format, 100 pmol of siRNA per well was transfected into the GH4C1 cells. After 6 h, the medium was changed to DMEM containing 10% FBS. Twenty-four hours after the first transfection, the transient transfection of TSHβ-Luc, Pit1 and GATA2 was performed using a calcium phosphate precipitation method. Sixteen hours after the second transfection, the medium was changed to DMEM supplemented with 10% FBS treated with AG1-X8 resin and activated charcoal. Cells were further incubated in the presence or absence of TRH. After twenty-four hours incubation, a luciferase assay and RNA extraction were performed.

### Electrophoretic Mobility Shift Assay (EMSA)

The EMSA was performed using a fragment of the radiolabeled POMC promoter containing typical NurRE (5′-gatcctagtgatatttacctccaaatgccagga-3′) and a fragment containing the human TSHβ bp −123∼−87 (5′-cagtatgaattttcaatagatgcttttcagataagaaa-3′), which has been reported to be responsible for TRH-induced stimulation [20], [32]. Double-stranded oligonucleotides were labeled with [α^32^P]dCTP by a fill-in reaction using a Klenow fragment of DNA polymerase I. NR4A1 was synthesized by *in vitro* transcription/translation from pcDNA3.1-NR4A1 using T_7_ RNA polymerase and the TNT-coupled reticulocyte lysate system (Promega Corporation). The binding reaction, gel electrophoresis, and autoradiography were performed under conditions described previously [50].

### Chromatin-immunoprecipitation (ChIP) Assay

ChIP assays were performed as we previously reported, using a kit from Upstate Biotechnology (Massachusetts, USA) [78]–[80]. GH4C1 cells were transfected with pA3TSHβ(−1192∼+37-Luc and incubated in medium containing 10% fetal bovine serum. After incubation overnight, 37% of formaldehyde was directly added to the culture at a final concentration of 1%, and the cells were incubated for 15 min at room temperature to crosslink protein to DNA. The whole cell extracts were pelleted and resuspended in 500 μl of lyses buffer (1% SDS/50 mM Tris-HCl, pH 8.1, 10 mM EDTA, 1 mM PMSF and 1 mg/ml aprotinin) for 10 min at 4°C. The lysate was sonicated 3 times with 10-sec pulses using a sonicator set at 70% of maximum power to reduce DNA length to between 200 and 1000 bp. The chromatin solution (500 μl) was used for each ChIP assay with 0.6 mg of a rabbit anti-NR4A1 antibody (LS-B114, LifeSpan BioScienes, Seattle, WA, USA). As a negative control, normal mouse IgG (sc-2025, Santa Cruz Biotechnology, CA, USA) was used. The primers used for the region between bp –138 and +13 were as follows, forward primer, 5′-GGTAAAGATATTGTGAGCTTGTTTGTCTAA-3′, and reverse primer, 5′- GCTGTGGTGACCCAAACTAAAAGC-3′, and the length of the predicted PCR product was 151 bp. The primers used for the region between bp -1192 and -1049 containing putative NurRE sequences (AAATATCA: -1091 bp and -1083 bp) were 5′-GGATCCCTTCCCTACACCATATAGAAAAAT-3′, and 5′-GTCATGAAATCTCTA CCCGGTCCTACATCT-3′, and the predicted size was 143 bp. Conventional PCR was performed in 50 μl with platinum high-fidelity (Invitrogen) for 30 cycles (annealing temperature of 60°C), and signals were stained with ethidium bromide in 2% agarose gels and scanned with a Molecular Imager FX (Bio-Rad). All ChIP assays were repeated at least three times.

### Statistical Analysis

Statistical analyses were performed with ANOVA and Student’s t test or the Wilcoxon/Kruskal-Wallis test using JMP (SAS Institute Inc., Cary NC).
